# The Utilization of Tunable Transducer Elements Formed by the Manipulation of Magnetic Beads with Different Sizes via Optically Induced Dielectrophoresis (ODEP) for High Signal-to-Noise Ratios (SNRs) and Multiplex Fluorescence-Based Biosensing Applications

**DOI:** 10.3390/bios12090755

**Published:** 2022-09-14

**Authors:** Chia-Ming Yang, Jian-Cyun Yu, Po-Yu Chu, Chia-Hsun Hsieh, Min-Hsien Wu

**Affiliations:** 1 Department of Electronic Engineering, Chang Gung University, Taoyuan City 33302, Taiwan; 2 Institute of Electro-Optical Engineering, Chang Gung University, Taoyuan City 33302, Taiwan; 3 Biosensor Group, Biomedical Engineering Research Center, Chang Gung University, Taoyuan City 33302, Taiwan; 4 Department of General Surgery, Chang Gung Memorial Hospital at Linkou, Taoyuan City 33302, Taiwan; 5 Department of Neurosurgery, Chang Gung Memorial Hospital at Linkou, Taoyuan City 33302, Taiwan; 6 Department of Materials Engineering, Ming Chi University of Technology, New Taipei City 23652, Taiwan; 7 Graduate Institute of Biomedical Engineering, Chang Gung University, Taoyuan City 33302, Taiwan; 8 Ph.D. Program in Biomedical Engineering, Chang Gung University, Taoyuan City 33302, Taiwan; 9 Division of Hematology/Oncology, Department of Internal Medicine, Chang Gung Memorial Hospital at Linkou, Taoyuan City 33302, Taiwan; 10 Division of Hematology/Oncology, Department of Internal Medicine, New Taipei Municipal Hospital, New Taipei City 23652, Taiwan; 11 Department of Chemical Engineering, Ming Chi University of Technology, New Taipei City 23652, Taiwan

**Keywords:** tunable transducer element, fluorescence, magnetic beads, ODEP, detection threshold

## Abstract

Magnetic beads improve biosensing performance by means of their small volume and controllability by magnetic force. In this study, a new technique composed of optically induced dielectrodphoresis (ODEP) manipulation and image processing was used to enhance the signal-to-noise ratio of the fluorescence for stained magnetic beads. According to natural advantages of size-dependent particle isolation by ODEP manipulation, biomarkers in clinical samples can be easily separated by different sizes of magnetic beads with corresponding captured antibodies, and rapidly distinguished by separated location of immunofluorescence. To verify the feasibility of the concept, magnetic beads with three different diameters, including 21.8, 8.7, and 4.2 μm, were easily separated and collected into specific patterns in the defined target zone treated as three dynamic transducer elements to evaluate fluorescence results. In magnetic beads with diameter of 4.2 μm, the lowest signal-to-noise ratio between stained and nonstained magnetic beads was 3.5. With the help of ODEP accumulation and detection threshold setting of 32, the signal-to-noise ratio was increased to 77.4, which makes this method more reliable. With the further optimization of specific antibodies immobilized on different-size magnetic beads in the future, this platform can be a potential candidate for a high-efficiency sensor array in clinical applications.

## 1. Introduction

Proteins are important factors in many biological processes, including reproduction of genomic information, gene expression regulation, mRNA transcription, metabolic catalytic reactions, and molecule transportation. In the past few decades [1], measurement of proteins and their concentrations in life sciences research and clinical diagnostics has been widely investigated by using enzyme-linked immunosorbent assays (ELISAs [2]), Western blots [3], protein microarrays [4], flow cytometry [5], lateral flow assays [6,7], and surface plasmon resonance (SPR) [8]. Protein detection techniques have been continuously improved in performance requirements such as sensitivity, specificity, dynamic range, multiplexing capabilities, reproducibility, ease of use, and cost. The major challenge is detecting multiple proteins at very low concentrations in complex biological samples (e.g., protein-based biomarkers in blood) for diagnosis and monitoring of disease. Currently, less than 10% of plasma proteins can be quantitatively detected with a high confidence level [9].

Before the simultaneous detection of multiple proteins in biological samples, high-throughput, low-cost, and low-volume sensing platforms with a sensing performance of high sensitivity and a low limit of detection (LOD) are desired [10,11]. However, a major challenge is interference from the nonspecific binding of nontarget proteins (e.g., background noise). Extra separation and purification techniques, such as centrifugation, desalting, filtration, and affinity depletion of other interfering molecules, were applied [12,13]. In the meantime, high-affinity reagents with a high selectivity to the target protein are required to reduce interference [14]. For a high efficiency and low cost of detection, the total volume of biological samples and reagents need to be reduced in real applications. There are new technologies engaged to satisfy these requirements, including the use of microfluidic design [15,16,17] and magnetic beads [18,19,20]. One of the most promising platforms is a digital ELISA method (e.g., called the single-molecule array (Simoa) proposed by David Walt’s group 2). With the help of magnetic beads and femtoliter-sized wells for single beads, the fluorescence generated by the enzyme–substrate reaction of a single molecule can be detected with several hundred-fold sensitivity compared to conventional ELISA [21]. This technology was proven in detection of SARS-CoV-2 protein [22], total tau- and S-100 calcium-binding protein of sports-related concussions [23], and cardiac troponin I [24]. In this platform, a high-resolution optical reader, special processed disc substrate composed of microwells for single magnetic beads, and a femtoliter volume for reagents are still necessary [2]. Furthermore, magnetic separation for immune sensors has been widely investigated for its high efficiency and fast response [25,26]. Immunomagnetic beads with a quantum dot-reversed assaying strategy have 50 times higher sensitivity than conventional methodology [27]. This kind of platform has been applied for analysis of circulating hepatocellular carcinoma cells [28], tumor cell subpopulations [26], foodborne pathogens [25], and influenza virus H9N2 [29]. An immunomagnetic reduction (IMR) assay was proven to detect total α-synuclein and neurofilament light chain (NFL) concentrations in plasma with the help of antibody-functionalized magnetic nanoparticles [30]. However, to achieve the abovementioned precision detection, an external high-density and tunable magnetic force was prepared in a high-cost facility.

Among these results, the limitation of simultaneous multiple sensing by means of magnetic beads still remains an issue. To keep the advantages of magnetic separation and explore further possibilities with affordable price and easy operation, we would like to explore the behavior of antibody-functionalized magnetic beads manipulated in a microfluidic channel by the optically induced electrokinetic (OEK), such as light-actuated AC electroosmosis (LACE) and optically induced dielectrodphoresis (ODEP) under certain operation conditions in the previous literature [19]. With the photosensitive semiconductor and specially designed illuminated light patterns, ODEP-based manipulation, as with the similar performance of dielectrophoresis (DEP) [31], can be easily achieved to control magnetic beads via a more flexible arrangement compared to conventional methods such as DEP and acoustofluidic methods [32]. In subsequent ODEP operations, collection and separation of magnetic beads with different diameters were demonstrated with specific dynamic light pattern designs. According to our experiences and constructed platform, a new concept that combines the natural advantages of magnetic beads and the strength of ODEP in microfluidics is proposed in this study to approach the desires of the real application of protein detection. ODEP and magnetic force could be both used to manipulate magnetic beads, which opens a possibility of combinational usage and applications. The stable and reliable methodologies proven in magnetic beads relative information can be easily applied to enhance sensing performance [8]. An investigation to check the fluorescence results of stained antibodies on various magnetic beads to construct multitunable transducer elements as a novel sensing array was systematically performed with the accumulation skill and image process with optimization of the detection threshold value of fluorescence images. Improvement of the signal-to-noise ratio for multisized magnetic beads is clearly presented in this work, which is a significant milestone for new biosensing methodology.

## 2. Materials and Methods

### 2.1. The Mechanism of ODEP for Microparticle Manipulation

ODEP has been proposed from the derivation of DEP by the replacement of physical metal electrodes with tunable and virtual optical-induced electrodes [33]. For ODEP-based microparticle manipulation, the nonuniform electric field is easily induced and controlled by the difference between surface potentials between areas, which can be used to polarize dielectric microparticles and generate the corresponding force on a polarized microparticle (i.e., ODEP manipulation force [34]). In general, a uniform electric field of the ODEP chip is first generated by applying an alternating current (AC) voltage between the top and bottom indium tin oxide (ITO) electrodes for the polarization of suspended dielectric microparticles in the solution. Then, a difference of applied voltage across the solution layer can be created by an extra illumination injected into the bottom photoconductive semiconductor layer due to the generation of electron–hole pairs excited by photons and following a significant reduction of electrical impedance. Areas with and without illumination automatically generate a locally nonuniform electric field that can be used to drive electrically polarized microparticles [35]. In various applications, including cells [36], polymer beads [37], and magnetic beads [18], dynamic light images can be easily arranged to manipulate microparticles in a controllable manner. A theoretical equation relative to dielectrophoresis (DEP) force is listed in Equation (1), which can also be applied to express the ODEP force generated on a microparticle [38]:

(1) $$F_{DEP}=2\pi r^{3}\varepsilon_{0}\varepsilon_{m}Re\left[f_{cm}\right]\nabla {\left|E\right|}^{2}$$

where *r*, $\varepsilon_{m}$, and ∇*E*^2^ represent radius of the microparticle, permittivity of the medium, and square of the electrical field gradient, respectively [39]. *Re*[*f_cm_*], which is determined by the angular frequency of the electric field (i.e., ω), permittivity and conductivity of the medium, and internal permittivity and conductivity of microparticle [40,41], is the key factor for the magnitude and operation direction of the ODEP force [42].

### 2.2. Materials

In this study, commercial streptavidin-coated magnetic microbeads commonly had different diameters (D) [i.e., D = 4.2 μm (SVM-40-10; SPHEROTECH, Lake Forest, IL, USA), with D = 8.7 μm (SVM-80-5; SPHEROTECH, Lake Forest, IL, USA) and 21.7 μm (SVM-200-4; SPHEROTECH, Lake Forest, IL, USA)] being used to investigate the manipulation of tunable transducer elements and corresponding fluorescence study. The structure of these magnetic microbeads has multiple layers from the polystyrene core to several outer layers, such as iron oxide, a protective polymer layer, and astreptavidin coating layer. In this study, these streptavidin-coated magnetic microbeads were prepared in diluted bovine serum albumin (BSA) buffer solutions (J885-5 L; PanReac AppliChem, Castellar del Vallès, BCN, Spain) with a volume ratio of 0.05%. The electrical conductivity was measured to be 6.5 μS/cm by a conductivity meter (C0510, Consort, Turnhout, Belgium).

### 2.3. Fabrication of ODEP Chips

**Figure 1:**
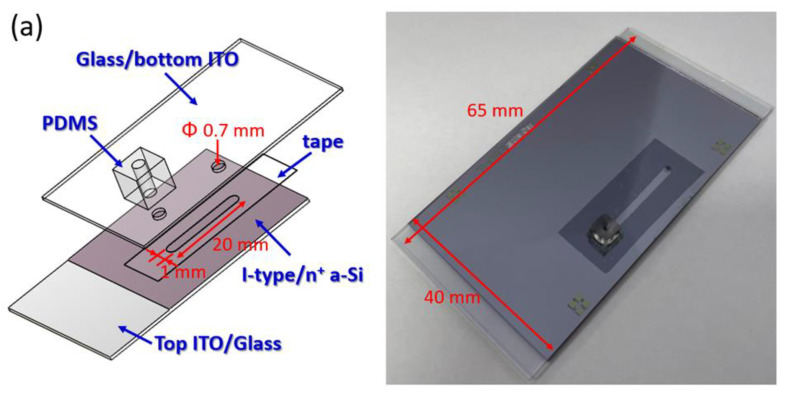

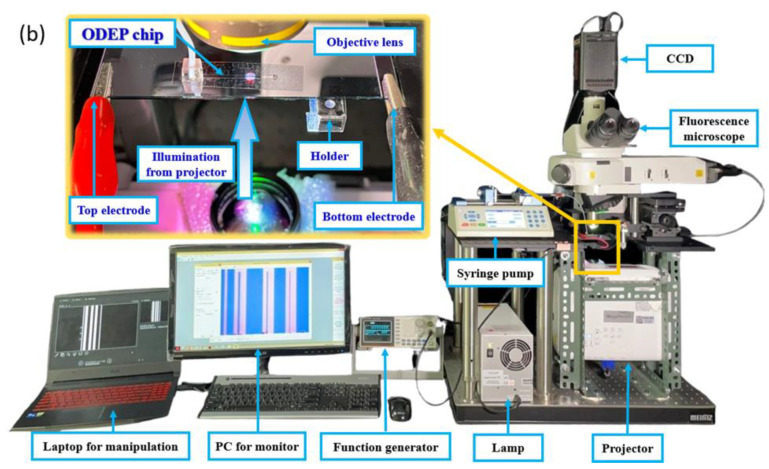
(**a**) Schematic plot and picture of the fabricated ODEP chip and (**b**) picture of the whole ODEP system setup.

ODEP chips were mainly constructed of a top conductive electrode of ITO/glass, a microchannel, and a bottom photoconductive semiconductor layer of a-Si:H/ITO/glass. The detailed process of the whole system is shown in our previous publication [19]. By following a previous design, the same structure of four stacked layers was fabricated by replacing a microchannel design, a new resource of top ITO/glass and a-Si:H/ITO/glass, which were fabricated by InnoLux Corporation (ZMI, Taiwan). The ODEP chip was composed of a polydimethylsiloxane (PDMS) connector, ITO/glass, adhesive tape with the microfluidics design of the microchannel layer, a major functional photoconductive substrate with an intrinsic hydrogenated amorphous silicon (a-Si:H) layer with a thickness of 1000 nm, and a heavily n^+^-doped hydrogenated amorphous silicon (n^+^ a-Si:H) layer with a thickness of 20 nm deposited by plasma-enhanced chemical vapor deposition (PECVD) (AKT4300, Applied Materials, Santa Clara, CA, USA) on an ITO/glass. The two through-holes on the top ITO/glass were fabricated by a driller machine to connect the microchannel layer as the inlet and outlet of the solution with magnetic beads in the following experiments and then fixed with a PDMS cubic connector by O_2_ plasma surface treatment for subsequent connection to the syringe pump. A designed structure of the microchannel and whole ODEP chip is schematically shown in Figure 1a. The microchannel was fabricated in double-sided adhesive tape (L298, Sun-yieh, TYN, Taiwan; thickness: 50 µm) defined by using a manual punch operation with a custom-made metal mold.

### 2.4. System Setup of ODEP Manipulation

Before operation, the fabricated ODEP chip was fixed to a self-designed chuck to have horizontal status and stable connections with the bias voltage supplement. The prepared magnetic bead suspension solution was loaded into the microchannel by using a syringe pump (Fusion 200, Chemyx, Stafford, TX, USA). To achieve the OEK-based manipulation of magnetic microbeads, a function generator (AFG-2125, Good Will Instrument Co., Ltd., NTPC, Taiwan) was used to apply an AC bias voltage between the top and bottom ITO conductive layer. A commercial digital projector (EB-X05, Epson, Nagano, Japan) controlled by a laptop was used to provide programmable light patterns to the backside of the ODEP chip for the manipulation of magnetic microbeads. A microscope, integrated with a charge-coupled device (CCD) (OPTEM-Zoom 160, Qioptiq inc., Fairport, NY, USA) and controlled by a personal computer, was set up to monitor the manipulation of magnetic microbeads from the top of the ODEP chips. For the fluorescence check, this microscope equipped with an excitation lamp (C-HGF1 130 W, NIKON, Tokyo, Japan) was fixed from the top side of the ODEP chip and filter lens. The exposure time and gain were set as 1000 ms and 4, respectively. During the manipulation and fluorescence monitoring, a light-shielding curtain was used to avoid interference ambient light. The whole ODEP system setup is shown in Figure 1b.

### 2.5. ODEP Manipulation of Magnetic Beads

In the evaluation of the basic manipulation of magnetic beads, three kinds of magnetic beads, as mentioned in Section 2.2, stained with a fluorescent dye were loaded into the microchannel of the microfluidic chip, as shown in Figure 1a, for the following procedures. First, to evaluate the performance of ODEP manipulation for magnetic beads, the maximum manipulation speed of each kind of magnetic bead was tested by the different moving speeds of a bar-type light pattern defined by the different moving times within a fixed distance. During the ODEP manipulation, bar-type light pattern-driven movements of magnetic beads were determined by the effective net force between the manipulation force generated on a magnetic bead and the viscous drag of the surrounding fluid. A drag force (*F_d_*) acting on a spherical particle in a continuous flow condition can be well explained by Stokes’ law, as listed in Equation (2) [43].

(2) $$F_{d}=6\pi \eta r\upsilon$$

where *r*, *η*, and *v* are the radius of a magnetic bead, viscosity of the fluid, and relative velocity between a magnetic bead and fluid, respectively. Therefore, due to the use of static fluid in this experiment, the drag force in a fixed solution and the same dimension of magnetic beads was determined mainly by the terminal velocity of magnetic beads manipulated by a bar-type light pattern. Then, the ODEP manipulation force generated on a magnetic bead can be simply evaluated as the drag force at the maximum speed of a moving light pattern applied in magnetic beads. The width of light pattern was selected as 100 μm for a stable manipulation [31]. The test procedure of the moving speed of a light pattern was started from 250 to 10 μm/s. With a higher speed, the higher drag force makes magnetic beads out of the control of the ODEP manipulation force generated by the light pattern. In our experiments, the maximum speeds of a moving light pattern for different kinds of magnetic beads were collected as the key parameter to separate and collect magnetic beads. As the second part of manipulation, various moving light patterns from a high-to-low speed were selected to separate magnetic beads from large-to-small dimensions in a self-designed procedure due to their size-dependent magnitudes of ODEP manipulation force, as demonstrated in Equation (1). After this separation and purification step, three transducer elements with three different diameters were easily constructed dynamically in the defined detection areas, named target zones. After collecting magnetic beads with the same diameter, a second collection step was designed to concentrate magnetic beads to a high density in a small area for subsequent fluorescence detection.

### 2.6. Fluorescence Treatments on Magnetic Beads and Images Processed by ImageJ Software

In this work, to quantitatively evaluate the collection efficiency and relative sensing performance, streptavidin-coated magnetic beads were bound with biotin-coated primary antibodies to mimic the clinical detection-used immunomagnetic microbeads. Streptavidin-coated magnetic beads were mixed with the prepared CD45 buffer solution (e.g., phosphate-buffered saline (PBS) with 2% fetal bovine serum (FBS) and 1 mM ethylenediaminetetraacetic acid/EDTA) of 100 μL and then mixed with the biotin-coated primary antibody solution (e.g., biotin-coated mouse anti-human CD45 antibodies in this study; Mouse IgG1, tcta30459, Taiclone Biotech Corp., TPE, Taiwan) of 1 μL for 10 min at 4 °C. The volume ratio between magnetic beads and primary antibodies was controlled from 1:1 to 2:1 for the experimental groups. After incubation, the sample was washed three times by using the abovementioned CD45 buffer solution to remove the unbound primary antibodies. After that, for subsequent image recognition and analysis, the abovementioned primary antibody-bound magnetic beads were immunofluorescently stained with 10-diluted Alexa Fluor 488-coated donkey anti-mouse IgG secondary antibodies (A-21202, Invitrogen, US) of 1, 2, or 4 μL for 1 h at 4 °C, which then displayed the green, fluorescent images in fluorescence microscopic observation with the excitation wavelength of 465–495 nm.

To achieve better pattern recognition, ImageJ software (version 1.53n 7, Wayne Rasband, NIH Retires, Bethesda, MD, USA) was used to process the digital signal of fluorescence images, including the color level, intensity, and corresponding area [44,45]. Due to the fluorescence of the green color staining by secondary antibodies, all images were filtered with green color for the digital level of its intensity. With the different setting of detection threshold value, the fluorescence images were transferred into 2-level images defined as a white and red colors of area, with value less and higher than the setting of a selective detection threshold value, respectively. Therefore, the area of all fluorescence spots can be further calculated by using ImageJ software, which can refer to the total intensity induced by the labeled magnetic beads. The illustration is presented in the results and discussion section below.

## 3. Results and Discussion

**Figure 2:**
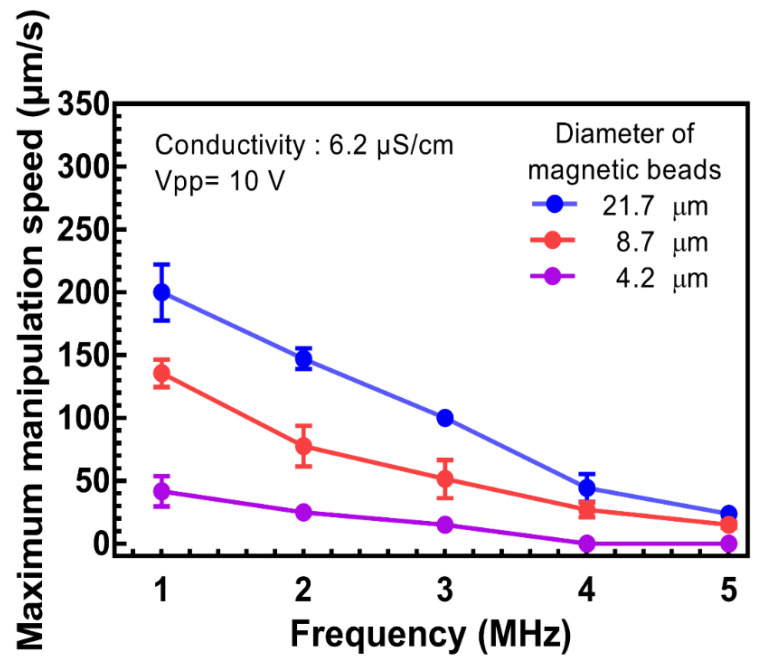
The maximum manipulation speed versus AC trigger frequency with a fixed Vpp of 10 V of magnetic beads with different diameters, including 4.2, 8.7, and 21.7 μm, in a 0.05% BSA solution.

First, the basic manipulation behavior of three different magnetic beads must be verified in advanced investigations. As mentioned in Section 2.5, the ODEP manipulation force can be simply evaluated as the drag force at the maximum speed of a moving light pattern applied in magnetic beads. The maximum manipulation speeds of all three kinds of magnetic beads were intensively studied for the AC frequency dependence in a fixed solution environment with conductivity of 6.2 μS/cm and a fixed AC bias peak-to-peak voltage of 10 V. As shown in Figure 2, the frequency was modified from 1 to 5 MHz for the normal ODEP operation region [46]. With a lower frequency (e.g., 800 kHz), the segregation of magnetic beads was found, and the manipulation behaviors were dramatically changed. A characteristic curve of the lower frequency and higher maximum manipulation speed can be fully explained by the *Re*[*f_cm_*] factor described in Equation (1) [40]. In the meantime, the larger diameter and higher maximum manipulation speed can be clearly understood by the positive correlation between the ODEP manipulation force and radius of the particle (*r*) described in Equation (1) [34]. Differences in the maximum manipulation speeds between three different magnetic beads can be observed at different frequencies. The behavior of maximum manipulation speed decreased with frequency matches to the cell manipulation [31]. For example, the lower the frequency, the wider the gap of the difference in the maximum manipulation speed that can be found, which is an opportunity for the separation of groups of magnetic beads with different diameters. To ensure a wider gap for a clear operation margin in real applications, the frequency at 1 MHz was selected for further manipulations in the following experiments.

**Figure 3:**
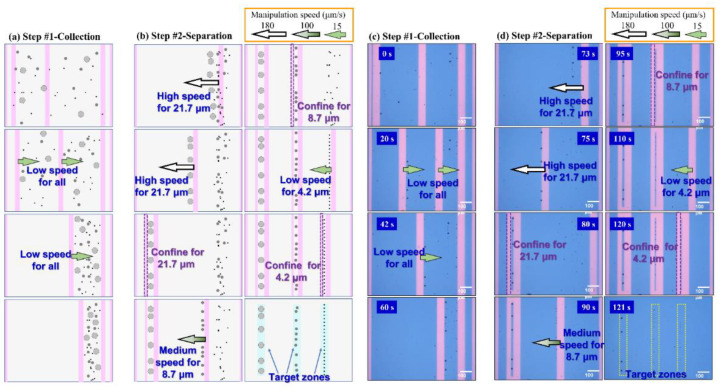
The procedure to generate three different transducer elements, individually constructed using three types of magnetic beads by selected manipulation speed of light patterns movements for two main steps including collection and separation, is shown by the schematic plot and real images captured from operation video: (**a**) schematic plots of Step #1 Collection, (**b**) schematic plots of Step #2 Separation, (**c**) real images of Step #1 Collection, (**d**) real images of Step #2 Separation. With different speeds of light patterns, magnetic beads with different diameters can be collected and separated to form their own line pattern as a tunable transducer element.

Based on the maximum manipulation speed of 1 MHz, a low speed of 15 μm/s of light pattern movement can be used to collect all magnetic beads, as shown in Figure 3a. It can be fully demonstrated that the ODEP manipulation force of our system setup can overcome the drag force at a manipulation speed of 15 μm/s. After the collection of all magnetic beads, a high speed of 180 μm/s of light pattern movement was chosen to push magnetic beads with a diameter of 21.7 μm only from all mixed magnetic beads to the left side as the first separation step, as shown in Figure 3b. This can be explained by the ODEP manipulation force generated on magnetic beads with a diameter of 21.7 μm, overcoming their drag force and then following the push of light pattern movement. This chosen high speed of 180 μm/s was higher than the maximum manipulation speeds of magnetic beads with diameters of 8.7 and 4.2 μm, respectively. This means that the ODEP manipulation forces generated on magnetic beads with a diameter of 8.7 or 4.2 μm cannot overcome the drag force generated at this high speed of 180 μm/s and then remaining constant. Then, fixed light patterns were used to confine all collected magnetic beads with a diameter of 21.7 μm to prevent potential disturbances in the following separation steps. With the same concept, a speed reduced to 100 μm/s of light pattern movement was used to collect magnetic beads with a diameter of 8.7 μm from the remaining mixed magnetic beads as the second separation step. All magnetic beads with a diameter of 4.2 μm were left after the light pattern moved away. Additionally, light patterns were generated to fix all magnetic beads with diameters of 8.7 μm. Finally, the low speed of 15 μm/s of light pattern movement was used to collect magnetic beads with a diameter of 4.2 μm and then fixed by light patterns. Three target zones with magnetic beads with specific diameters were obtained, as shown in the final step. The video of the whole manipulation procedure is shown in the supplemental material named “Construct transducer elements with filtered magnetic beads”. Captured images from CCD at various times for Step #1—Collection—and Step #2—Separation—are shown in Figure 3c,d, respectively. Schematic arrows with different lengths and colors are marked in the illustrations to describe the moving direction and speed of the light bar. It can be clearly seen that two light bars with the same low speed (e.g., 15 μm/s) were used to push all magnetic beads to the right side, regardless of their diameter, simultaneously. Then, the second part of manipulation (e.g., Step#2-Separtion) was performed, with a light bar with a speed of 180 μm/s to push magnetic beads with a diameter of 21.7 μm to the left side from 73 to 80 s. Then, a second light bar was applied to prevent all collected magnetic beads at 80 s from slipping away. A light bar with a speed of 100 and 15 μm/s to push magnetic beads with diameters of 8.7 and 4.2 μm to the left side can be seen in images at 90 and 110 s, respectively. Finally, three target zones for magnetic beads filtered by different diameters can be seen in the image at 121 s. Due to the repulse force between the polarized magnetic beads, the distances between magnetic beads in the target zones are automatically generated; these can be improved with further manipulation, named accumulation, in the following part.

**Figure 4:**
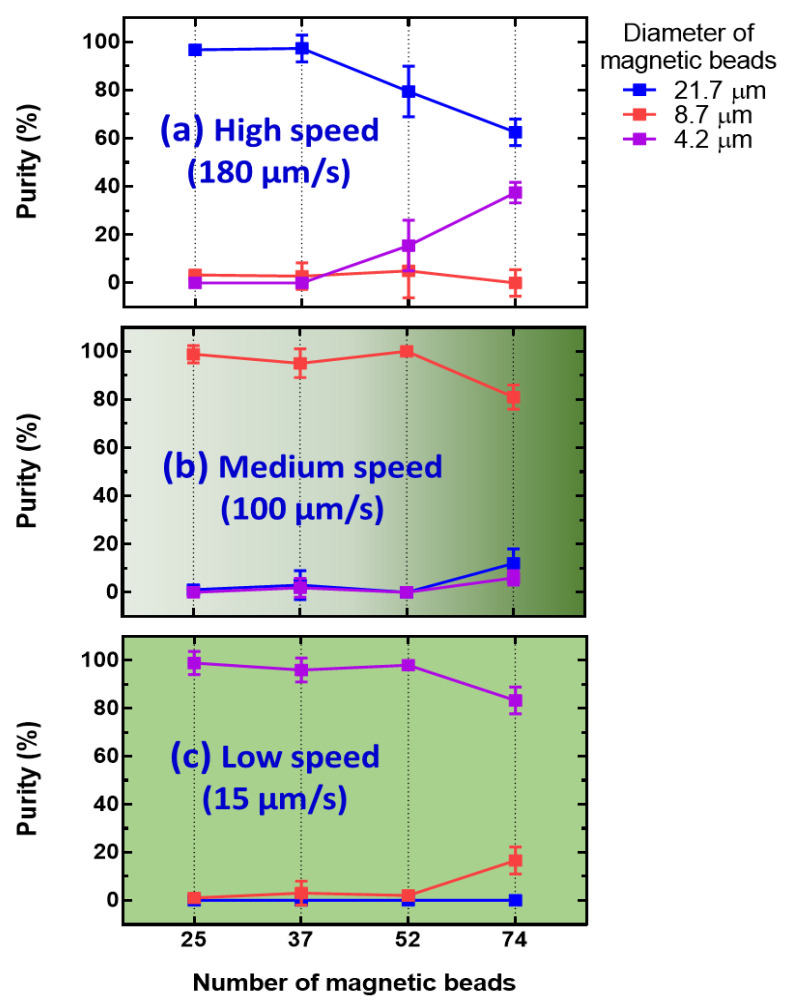
The purity of separation results for magnetic beads mixed with three different diameters and various total numbers by using speed sequence during the manipulation of (**a**) 180, (**b**) 100, and (**c**) 15 μm/s.

Before the accumulation procedure was undertaken, the maximum number of magnetic beads collected in the ODEP manipulation was checked by using different numbers of magnetic beads of 25, 37, 52, and 74 with three different diameters. The procedure, demonstrated in Figure 3c,d, was performed to evaluate the possibility of a high density of magnetic beads in the target zone. Then, the separation collection efficiency (e.g., named “Purity”) was calculated as the ratio between the number of each kind of magnetic bead to the total number after the specific manipulation speed, including 180, 100, and 15 μm/s, in their target zone. The calculated purities are drawn according to the total number of magnetic beads of 25, 37, 52, and 74, as shown Figure S1a–d, respectively. A purity > 85% was observed for a total number of 25 and 37, as shown in Figure S1a,b, respectively. This result can be explained by the lower aggregation of magnetic beads and subsequent very low disturbance. When the total number was increased to 52, the purity after a high manipulation speed was decreased, as shown in Figure S1c. This could be due to the higher aggregation for small magnetic beads making the particle size increase and following a large ODEP manipulation force. Once the total number of magnetic beads increased to 74, the purity further decreased, as shown in Figure S1d. Therefore, a higher number is not preferred in this kind of manipulation for the purpose of purification. For a clear comparison, the dependence of purity after different separation steps is redrawn for high (e.g., 180 μm/s), medium (e.g., 100 μm/s), and low (e.g., 15 μm/s) speeds, as shown in Figure 4a–c, respectively. For the high collection purity of magnetic beads, a manipulation speed of 180 μm/s was defined for magnetic beads with a diameter of 21.7 μm and a total number < 37, as shown in Figure 4a. With the sequence of manipulation speed from high-to-low, manipulation speeds of 100 and 15 μm/s were used for total numbers <52 magnetic beads with diameters of 8.7 and 4.2 μm, respectively. Based on this finding, it is difficult to both have a large number (e.g., only workable in total number less than 37 for 3 kinds of magnetic beads in this study) and high purity of magnetic beads with the same diameter (e.g., purity deceases with total number increases) after ODEP manipulation at once. Therefore, a further improvement of the accumulation step is designed to raise the number and density in a certain area of magnetic beads in the following section.

**Figure 5:**
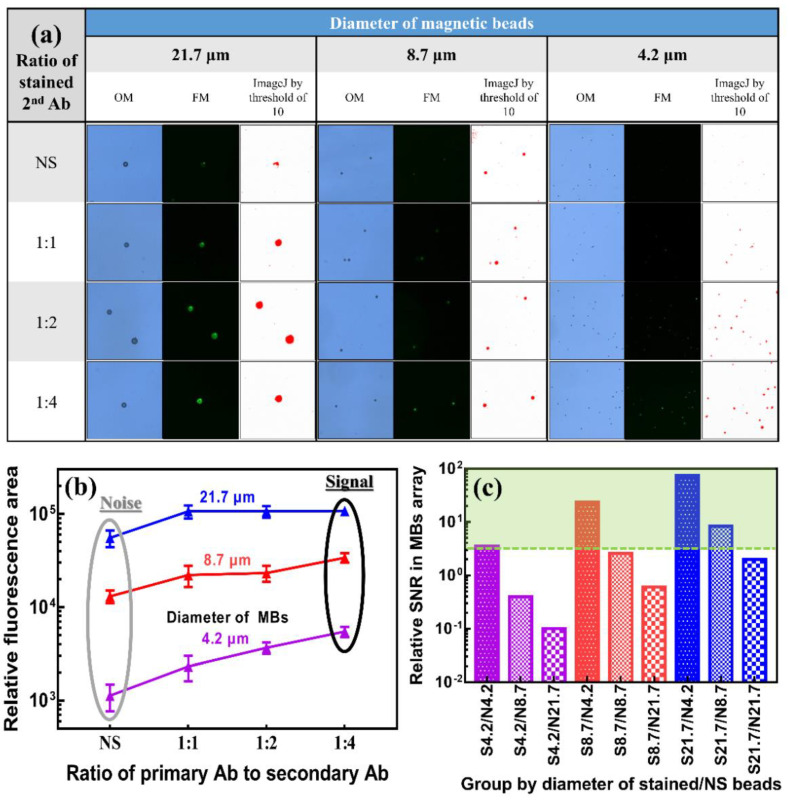
(**a**) Images under an optical microscope (OM), fluorescence microscope (FM), and ImageJ-processed with a detection threshold of 10. (**b**) Relative fluorescence area after filtering by ImageJ for magnetic beads with different diameters, including 21.7, 8.7, and 4.2 μm, and different staining ratios between primary and secondary antibodies, including NS, 1:1, 1:2, and 1:4. (**c**) Relative SNR between the fluorescence area of stained and NS magnetic beads for different combinations of bead sizes.

Before manipulating immunostained magnetic beads, different staining ratios between the primary and secondary antibodies were used to verify the fluorescence response of magnetic beads with different diameters. Images for different magnetic beads with different stained ratios captured by optical microscope (OM), fluorescence microscopy (FM), and ImageJ-processed from FM were collected, as shown in Figure 5a, for a clear comparison. As shown in Figure 5a, autofluorescence was observed for the nonstained (e.g., named “NS”) group, especially in the large magnetic beads (e.g., 21.7 μm). This behavior can be referred to as the background noise level of the fluorescence-based sensing method. As the ratio of secondary antibodies increased, the intensity and area of fluorescence increased for all three kinds of magnetic beads, which matched the general expectation. To obtain a better quantitative analysis, ImageJ software was used to process fluorescence images with a green color filter for a detection threshold of 10, and the stained area was remarked red. For a clear quantitative comparison, the relative fluorescence area higher than a detection threshold of 10 was calculated by ImageJ for magnetic beads (e.g., magnetic beads are named “MBs” in the figure) with different diameters and different stained ratios between primary and secondary antibodies, which is replotted as shown in Figure 5b. As the added volume of secondary antibodies increased, the relative fluorescence area increased. For the autofluorescence of NS groups, which refers to background noise in the potential test environment with multiple kinds of magnetic beads, the relative fluorescence area also increases with the diameters of the magnetic beads. Even the relative fluorescence area of the NS group for magnetic beads with a diameter of 21.7 μm was higher than that of the group with a staining ratio between primary and secondary antibodies of 1:4 for beads with diameters of 8.7 and 4.2 μm, which may be a concern in real applications. To further explore the interference level of magnetic beads with various diameters, a relative signal-to-noise ratio (SNR) was defined as the relative fluorescence area of the stained group divided by that of the NS group (e.g., “signal” from the stained group and “noise” from the NS group). To better understand the relative SNR in arrays with three kinds of magnetic beads, all combinations of three different sizes of magnetic beads stained with primary antibodies to secondary antibodies at a ratio of 1:4 were plotted versus three different sizes of NS magnetic beads based on the data shown in Figure 5b. As shown in Figure 5c, the relative SNRs of all combinations of three magnetic beads with diameters of 21.7, 8.7, and 4.2 μm are clearly presented for comparison. Based on the minimum requirement of the SNR, an SNR value < 3 is not acceptable for real applications [47]. Once the level and area of fluorescence are not high enough, the autofluorescence effect (e.g., noise level) of NS magnetic beads easily induces a low SNR, especially in large magnetic beads. As shown in Figure 5c, all groups with NS beads with a diameter of 21.7 μm have an SNR < 3. The SNR between the stained and nonstained magnetic beads with a diameter of 4.2 μm is only 3.5, which suggested a need to improve before real applications. In the meantime, the smaller stained magnetic beads were more easily impacted by the NS magnetic beads. This limited performance observed in small stained magnetic beads is inferior to having a wide detection range and very low limit of detection with a strong evidence level for further sensing applications based on our current setting. Before the minimization of the autofluorescence effect of large NS magnetic beads, some experiments are performed to enhance the signal of small stained magnetic beads by the collection and accumulation of small magnetic beads through ODEP manipulation.

**Figure 6:**
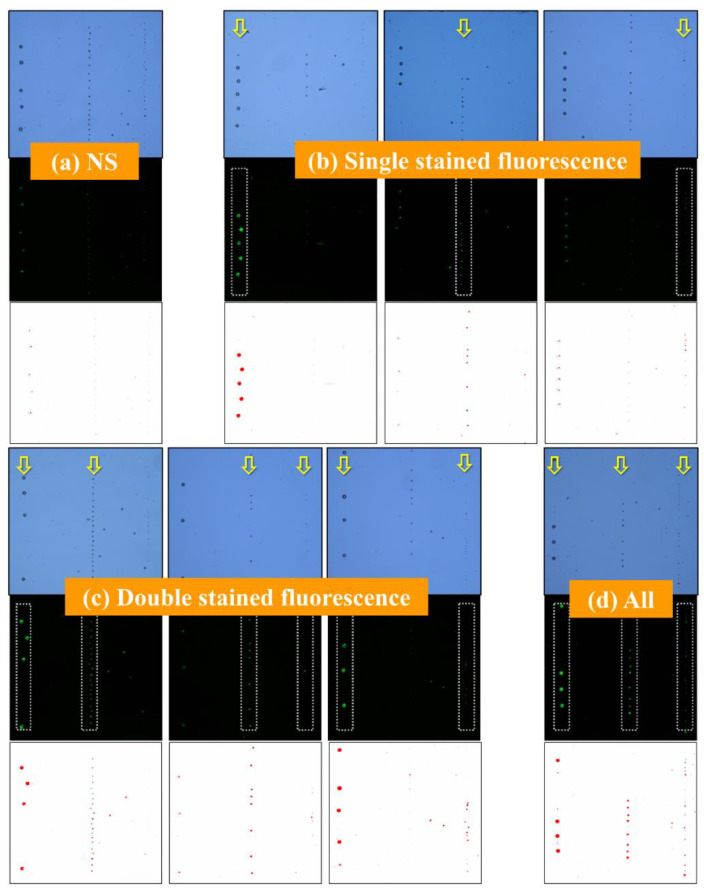
Comparison of optical and fluorescence images, and images filtered by ImageJ software with a detection threshold of 10 for magnetic beads mixed with three different diameters and stained with a ratio of the primary and secondary antibody of 1:4, including (**a**) 0-, (**b**) 1-, (**c**) 2-, and (**d**) 3-stained groups with different diameters for all combinations. It can be clearly seen that three groups with different diameters were easily separated in light images, and only the labeled groups can be observed in fluorescence images.

To evaluate the possibility of fluorescence images for multiple sensing targets binding on magnetic beads, ODEP manipulation followed with the procedure described in Figure 3a was applied on all combinations of different types of magnetic beads stained with a ratio of the primary and secondary antibody of 1:4 to collect corresponding fluorescence images for three separate target zones composed of three tunable transducer elements after dynamic collection in this part. In addition, there was no clear difference in the manipulation speed for stained and NS magnetic beads, which can be concluded from the lack of clear changes in the radius of magnetic beads and a similar polarization effect under ODEP manipulation. First, three target zones with NS magnetic beads separated by different diameters were clearly observed in OM images, as shown in Figure 6a. All magnetic beads were not stained, but their fluorescence responses were still found. Clear autofluorescence effects on magnetic beads with a diameter of 21.7 μm can be considered a source of potential noise during the fluorescence evaluation for all combinations in multiple transducer elements, which is similar to the results of individual and spreading beads, as shown in Figure 5a. To further check the collection results and corresponding fluorescence, a single type of magnetic bead was stained and then mixed with the other two NS magnetic beads to run the same ODEP manipulation procedure. Therefore, a total of three results for fluorescence-stained magnetic beads with three different diameters are presented together, shown in Figure 6b. Yellow arrows are marked to the target zone for fluorescence-stained magnetic beads with the same diameter. In the three target zones in the three sets of figures, the autofluorescence of magnetic beads with a diameter of 21.7 μm is significantly higher than that of small stained magnetic beads, as shown in the 2nd and 3rd conditions, which is confirmed to be a high-level noise in the direct data processing. To have a better recognition in this kind of array, a database with the relative fluorescence results of the negative control group, which is not friendly for usage, should be prepared in advance, as shown Figure 6a. Then, fluorescence images for the magnetic beads with a diameter of 4.2 μm could possibly be distinguished with the extra effort, which is less efficient in real applications.

In the meantime, the spatial resolution in the photodetector of FM could also be an important factor impacting the sensing results, especially in small magnetic beads. As shown in Figure 6c, two types of magnetic beads were stained and mixed with the other NS magnetic beads for the same separation procedure. Due to the same autofluorescence of magnetic beads with a diameter of 21.7 μm, the images are quite similar for the three groups, as shown in the 2nd condition of Figure 6c. Without the help of ODEP manipulation to generate the target zone, the multiple fluorescence sensing with the same color filter makes it difficult to obtain fluorescence images with a high confidence level. Finally, all three types of magnetic beads were stained, and the clear difference between the three target zones can be easily checked, as shown in Figure 6d. With the help of ODEP manipulation, three tunable transducer elements are easily generated for magnetic beads with the same diameter, which can be treated as three target zones for FM evaluation in this proposed methodology. In terms of having a valid sensing result of all possible conditions, the current method still has issues in applications and the same labeling color occurs only in one or two magnetic beads due to the noise of the autofluorescence of large magnetic beads. In summary, the necessity of an extra database with negative control samples (e.g., NS groups), the autofluorescence of large magnetic beads, the low signal level of small magnetic beads, and the limitation of image resolution in FM images could be the constraints of this kind of methodology for real sensing applications.

**Figure 7:**
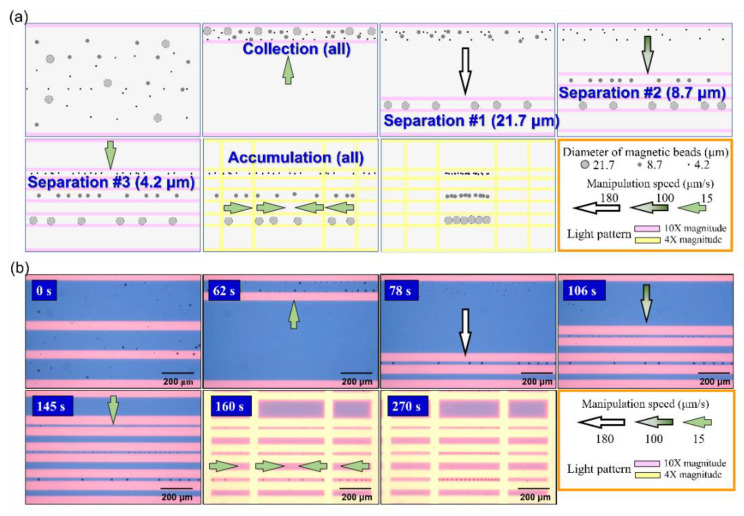
(**a**) The schematic plot and (**b**) real images captured from video of multiple manipulations including collection, separation, and accumulation of magnetic beads with three different diameters to form tunable and concentrated transducer elements for fluorescence enhancement. To collect more magnetic beads to form three transducer elements, all with a higher intensity, a wide collection area of light pattern is obtained by changing an object lens with magnification from 10× to 4×, as shown in the images captured at 160 and 270 s.

To enhance the low SNR (e.g., SNR < 3) in the group of small stained magnetic beads with a diameter of 4.2 μm, a new skill using a further manipulation step, called accumulation, was used to enhance the signal by collecting more magnetic beads in a wider area and then concentrating them 3-fold in a certain target zone. The detailed ODEP manipulation procedure of this step is shown in Figure 7a. Due to the limitation of this system constructed by commercial facilities, the manipulation area is limited by the projecting image with a ratio of 16:9, which means that a large dimension in the X-axis can be utilized to collect magnetic beads compared to the Y-axis in the previous experiment shown in Figure 3a. In the meantime, the magnification of the object lens is also changed from 10× to 4×, which has a large field of view for the ODEP manipulation. With the same procedure of separation of three types of magnetic beads from the bottom to the top area, four extra vertical light bar patterns with a speed of 15 μm/s were applied from the outer area to push collected magnetic beads to the center of the vision zone simultaneously. The video of the whole manipulation procedure is shown in the supplemental material named “Densified transducer elements with 3 different magnetic beads by accumulation procedure”. Captured images of all key steps from the video are shown in Figure 7b. The magnification of the object lens was changed from 10× to 4× after 160 sec and follows background color changes due to the higher light intensity collected. Three tunable transducer elements composed of magnetic beads of three different diameters with a 3-fold-high density can be obtained with this enhanced ODEP manipulation as a function of accumulation. Due to the interference of the background autofluorescence effect, a further study of the adjustment of the detection threshold value in ImageJ software was applied to screen the impact of this kind of noise. Two fluorescence images in the target zone with accumulated magnetic beads with a diameter of 4.2 μm with and without labeling were collected and processed with different detection threshold values in ImageJ software (version 1.53n 7, Wayne Rasband, NIH Retires, Bethesda, MD, USA). 

**Figure 8:**
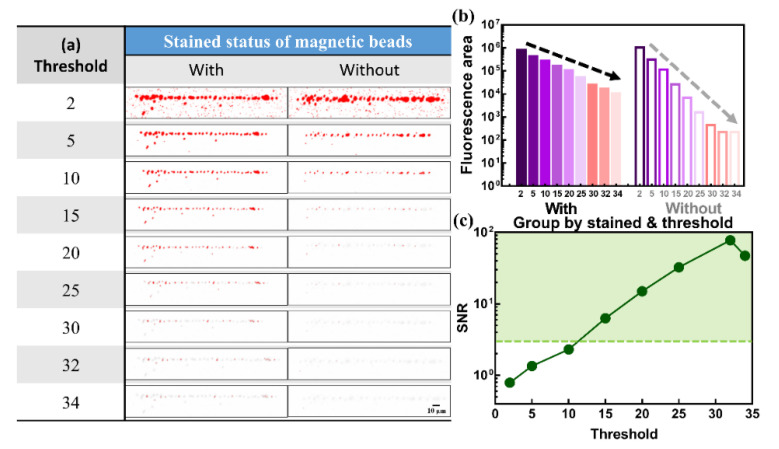
(**a**) Images and (**b**) bar chart of the relative fluorescence area and (**c**) SNR calculated by ImageJ with a detection threshold of 2, 5, 10, 15, 20, 25, 30, 32, and 34 magnetic beads with a diameter of 4.2 μm with and without staining. It can be clearly seen that the detection threshold value of 32 is a good index to distinguish magnetic beads with and without staining.

As shown in Figure 8a, two sets of images obtained by using ImageJ software with detection threshold values of 2, 5, 10, 15, 20, 25, 30, 32, and 34 were collected to compare the differences. The whole fluorescence image is transferred to the different intensity values by the green color filter, which means that the area with white color is defined as having no fluorescence response. Then, the area with red and light gray color is defined as the area with fluorescence intensity value higher and lower than the detection threshold value. At the detection threshold of 0 and 1, no shape of magnetic beads was found in the area full of red color (figure is not shown here). Area with red color for the NS group are very similar to the group with labeling by using a low detection threshold value (e.g., 2, 5 and 10), which is not acceptable for real applications due to interference of the autofluorescence of magnetic beads. However, the red area for the NS group (e.g., caused by autofluorescence and defined as the noise level) decreases more compared to the red area of the stained group (e.g., stained fluorescence defined as the signal level) when the detection threshold value increases, which may have a better SNR in real applications. To obtain a better comparison, the calculated fluorescence area with filtering of different detection threshold values for magnetic beads with and without stain is plotted, as shown in Figure 8b. With detection threshold values of 0 and 1, no clear image of magnetic beads can be found owing to the interference of background noise. Stained magnetic beads have a larger fluorescence area than NS magnetic beads when detection threshold values are higher than two. The fluorescence area decreases as the detection threshold value increases. The slope of the decreasing fluorescence area by the detection threshold value was higher in the NS group. To find an optimized point, the SNRs are calculated as the ratio of the fluorescence area screened by the specific detection threshold value between magnetic beads with and without stain, as shown in Figure 8c. The SNR clearly increases with the detection threshold value. The lowest and highest SNR values of 0.79 and 77.4 were found at detection thresholds of 2 and 32, respectively. Then, the SNR decreases slightly at a detection threshold of 34, which may be because only the signal was screened for but not the noise level, indicating that all noise from the NS group is thoroughly eliminated and that the detected signal from stained magnetic beads gradually decreases when the detection threshold exceeds 32. In this accumulated line-type transducer element composited by magnetic beads with a diameter of 4.2 μm, the optimized detection threshold value can be suggested as 32 with 98-, 57.3- and 33.7-fold increments of SNR compared to detection threshold values of 2, 5, and 10, respectively. With the dramatic improvement in SNR by ODEP manipulation and detection threshold value adjustment for magnetic beads with a diameter of 4.2 μm, simultaneous detection with multisized magnetic beads functionalized with antibodies can be proven to be a high-potential biosensing platform with the advantages of high efficiency, multisensing, high sensitivity, and low sample volume requirements.

## 4. Conclusions

We successfully demonstrated a new technique composed of ODEP manipulation and image processing to enhance the signal-to-noise ratio of the fluorescence for stained magnetic beads. Moreover, under ODEP manipulation, three dynamic transducer elements constructed by magnetic beads with different diameters can be easily obtained to increase the efficiency of biomarker collection and reduce the volume requirement of clinical samples, and which can be used as a tunable and efficient sensor array. Magnetic beads with three different diameters, including 21.8, 8.7, and 4.2 μm, are easily separated and collected into specific patterns in the defined area, which can be easily used to investigate the fluorescence of the stained results. The SNR between the stained and nonstained magnetic beads with a diameter of 4.2 μm is 0.79 and then increases to 77.4 by means of accumulation by ODEP and setting detection thresholds which range from 2 to 32. With the completion of this study, the confidence level of real application can be significantly improved. With the careful design and optimization of specific antibodies immobilized on different-size magnetic beads in the future, this developed platform can be further applied in sensor arrays in clinical applications with high efficiency.

## Data Availability

Not applicable.

## Supplementary Materials

The following supporting information can be downloaded at: https://www.mdpi.com/article/10.3390/bios12090755/s1, Figure S1: The purity of separation for volume magnetic beads with three different diameters and various total numbers, Video S1: Construct transducer elements with filtered magnetic beads, Video S2: Densified transducer elements with 3 different magnetic beads by accumulation procedure.
