# Supplementary material for: The Utilization of Tunable Transducer Elements Formed by the Manipulation of Magnetic Beads with Different Sizes via Optically Induced Dielectrophoresis (ODEP) for High Signal-to-Noise Ratios (SNRs) and Multiplex Fluorescence-Based Biosensing Applications
Source: Biosensors (Basel). 2022 Sep 14;12(9):755. doi: 10.3390/bios12090755 (PMC9496456; doi:10.3390/bios12090755)
Supplement: Supplementary file 1 [file biosensors-12-00755-s001.zip › Supplementary Materials.pdf]

biosensors

MDPI

Article

The utilization of tunable transducer elements formed by the manipulation of magnetic beads with different sizes via optically induced dielectrophoresis (ODEP) for high signal-to-noise ratios (SNRs) and multiplex fluorescence-based biosensing applications

Chia-Ming Yang [1,2,3,4,5,6], Jian-Cyun Yu [7], Po-Yu Chu [8], Chia-Hsun Hsieh [9,10], and Min-Hsien Wu [7,8,9,10,11,*]

[1] Department of Electronic Engineering, Chang-Gung University, Taoyuan City 33302, Taiwan
[2] Institute of Electro-Optical Engineering, Chang Gung University, Taoyuan City 33302, Taiwan
[3] Biosensor Group, Biomedical Engineering Research Center, Chang Gung University, Taoyuan City 33302, Taiwan
[4] Department of General Surgery, Chang Gung Memorial Hospital at Linkou, Taoyuan City 33302, Taiwan
[5] Department of Neurosurgery, Chang Gung Memorial Hospital at Linkou, Taoyuan City 33302, Taiwan
[6] Department of Materials Engineering, Ming-Chi University of Technology, New Taipei City 23652, Taiwan
[7] Graduate Institute of Biomedical Engineering, Chang Gung University, Taoyuan City 33302, Taiwan
[8] Ph.D. Program in Biomedical Engineering, Chang Gung University, Taoyuan City 33302, Taiwan
[9] Division of Hematology/Oncology, Department of Internal Medicine, Chang Gung Memorial Hospital at Linkou, Taoyuan City 33302, Taiwan
[10] Division of Hematology/Oncology, Department of Internal Medicine, New Taipei Municipal Hospital, New Taipei City 23652, Taiwan
[11] Department of Chemical Engineering, Ming Chi University of Technology, New Taipei City 23652, Taiwan
* Correspondence: mhwu@mail.cgu.edu.tw; Tel.: +886-3-2118-800 (ext. 3599)

Citation: Yang, C.-M.; Yu, J.-C.; Chu, P.-Y.; Hsieh, C.-H.; Wu, M.-H. The Utilization of Tunable Transducer Elements Formed by the Manipulation of Magnetic Beads with Different Sizes via Optically Induced Dielectrophoresis (ODEP) for High Signal-to-Noise Ratios (SNRs) and Multiplex Fluorescence-Based Biosensing Applications. *Biosensors* **2022**, *12*, 755. https://doi.org/10.3390/bios12090755

Received: 5 August 2022
Accepted: 11 September 2022
Published: 14 September 2022

Publisher's Note: MDPI stays neutral with regard to jurisdictional claims in published maps and institutional affiliations.

Copyright: © 2022 by the authors. Submitted for possible open access publication under the terms and conditions of the Creative Commons Attribution (CC BY) license (https://creativecommons.org/licenses/by/4.0/).

Supplementary Figure

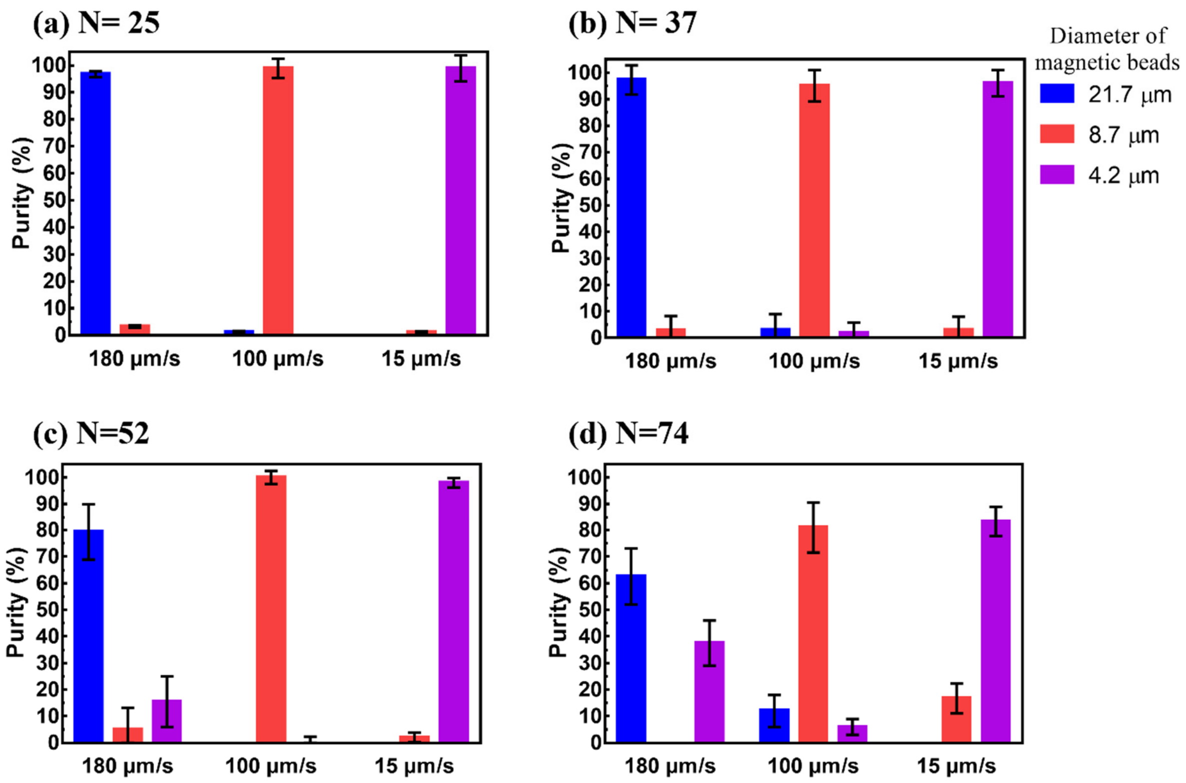

Figure S1. The purity of separation for volume magnetic beads with three different diameters and various total numbers of **(a)** 25, **(b)** 37, **(c)** 52, and **(d)** 74 by using manipulation speeds of 180, 100, and 15 μm/s, respectively.

Supplementary Video

Video S1: Construct transducer elements with filtered magnetic beads

Video S2: Densified transducer elements with 3 different magnetic beads by accumulation procedure
